# Transpalpebral Electrical Stimulation as a Novel Therapeutic Approach to Decrease Intraocular Pressure for Open-Angle Glaucoma: A Pilot Study

**DOI:** 10.1155/2018/2930519

**Published:** 2018-07-19

**Authors:** Félix Gil-Carrasco, Daniel Ochoa-Contreras, Marco A. Torres, Jorge Santiago-Amaya, Fidel W. Pérez-Tovar, Roberto Gonzalez-Salinas, Luis Nino-de-Rivera

**Affiliations:** ^1^Glaucoma Department, Hospital Luis Sánchez Bulnes, Asociación para Evitar la Ceguera en México I.A.P, Mexico City, Mexico; ^2^Artificial Vision Laboratory, Instituto Politécnico Nacional, Mexico City, Mexico; ^3^Research Department, Asociación para Evitar la Ceguera en México I.A.P, Mexico City, Mexico

## Abstract

**Purpose:**

To determine the effect on intraocular pressure of transpalpebral specific exogenous voltages in a cohort of open-angle glaucoma patients.

**Methods:**

This is a prospective, comparative, and experimental pilot study. The electrical stimuli applied consisted of 10 Hz, biphasic, nonrectangular current pulses (100 *μ*A) delivered from an isolated constant current stimulator. At intake, baseline IOP measurements were obtained from each eye. The measurement was repeated before and after microstimulation until the end of the treatment.

**Results:**

Seventy-eight eyes of 46 patients diagnosed with POAG were studied: 58 eyes with maximum tolerated medical treatment and 20 eyes without treatment (naïve). The mean baseline IOP on the treated POAG group was 19.25 mmHg ± 4.71. Baseline IOP on the naïve group was 20.38 mmHg ± 3.28. At the four-month follow-up visit, the mean IOP value on the treatment group was 14.41 mmHg ± 2.06 (*P* < 0.0001). The obtained mean IOP measurement on the treatment-naïve group was 15.29 mmHg ± 2.28 (*P* < 0.0001).

**Conclusions:**

The hypotensive response obtained using transpalpebral electrical stimulation on POAG patients, both on treatment-naïve patients and on patients receiving maximum tolerable treatment, was statistically significant when comparing basal IOP measurements to those obtained at the four-month follow-up visit.

## 1. Introduction

Primary open-angle glaucoma (POAG) is considered the world's leading cause of irreversible blindness [1]. One of the main risk factors for the occurrence and progression of this disease is an increase in intraocular pressure (IOP), primarily due to the dysfunction of the conventional drainage or trabecular pathway [2]. Oxidative stress and vascular damage play major roles in triggering apoptotic cell loss in these tissues [3, 4]. Molecular alterations occurring in the ocular anterior chamber during the early course of glaucoma trigger this cell loss [5]. Therefore, initial treatments, such as hypotensive drugs, usually aim to lower IOP. These drugs primarily focus on decreasing aqueous humor (AH) production or, alternatively, improving the outflow via nonconventional routes. Parasympathetic mimetic drugs can indirectly improve AH depletion via the trabecular route by stimulating muscular fibers contractility in the trabecular meshwork, which improves the AH outflow [5]. The trabecular meshwork is not merely a passive drainage filter but acts directly and actively on the resistance to AH passage via mechanisms that are not fully understood [6].

Several authors have described alterations in the mechanical properties of trabecular cells due to ion channel dysfunction, which affects the volume and elasticity of the cell. These changes may affect the permeability or ease of drainage of the trabecular system, and the contractile state and its induction have been recently studied in this context [7]. Stumpff et al. demonstrated that abnormal deposits of tyrosine kinase in the trabecula are related to the dysfunctional passage of the AH through the trabeculae into Schlemm's duct. These abnormal deposits cause the trabecular cell to increase in volume and lose elasticity, primarily due to the dysfunction of calcium- (Ca^2+^-) activated potassium (maxi K) channels [8]. Moreover, tyrosine kinase inhibitors activate the maxi-KCa^2+^ (BKCa^2+^) channels in the bovine trabecular veins and that hyperpolarization caused by the potassium efflux of the trabecular cells can lead to the relaxation of the trabecular meshwork to facilitate the outflow and passage of AH to Schlemm's channel [8–10]. Previous reports showed that tyrosine kinase inhibitors relax the bands of precontracted trabecular tissue. Specifically, the application of *genistein*, a tyrosine kinase inhibitor in trabecular cells previously contracted with acetylcholine, induced a dose-dependent reversible increase in the output current to 578% ± 154% (*n*=16) of the initial current levels [1, 9]. The electrophysiology of BKCa^2+^ channels observed when inhibiting tyrosine kinase demonstrates their activation and their function in the regulation of intracellular Ca^2+^. Soto et al. showed that the concentrations of intracellular Ca^2+^ in trabecular meshwork cells (TMCs, human, and bovine) directly correlated with the magnitude of the ionic current of BKCa^2+^ [11]. Furthermore, the behavior of the voltage-dependent K^+^ channels in TMCs is of the current rectifier type and directly correlates with increasing concentrations of intracellular Ca to modify the membrane potential [9, 10].

Recent studies by our research group [12] and in the Andreas Schattz group [9] demonstrate that transcorneal electrical stimulation (TES) using biphasic pulses of 20 Hz and up to 1100 *µ*A are beneficial to patients with retinitis pigmentosa (RP). Moreover, we noted that the application of TES in patients with PR decreased the IOP in several of these patients, which prompted us to study the effects of electrical stimulation as a possible alternative to control IOP.

The present pilot study aims at demonstrating a significant IOP decrease employing transpalpebral electrical stimulation (TPES) on open-angle glaucoma patients with maximum tolerated medical treatment and on those without treatment (naïve patients).

## 2. Materials and Methods

The Internal Review Board of the Asociación para Evitar la Ceguera en México I.A.P. approved this study. All the procedures conformed to the tenets of the Declaration of Helsinki; the good clinical practices guidelines comply with the issued regulations of the Standards for Privacy of Individually Identifiable Health Information (HIPAA). A signed written informed consent form was obtained from all of the participants after providing an explanation of the procedures to be used and possible complications.

### 2.1. Design

This is a prospective, comparative, and experimental study, conducted at the Glaucoma Department at the Asociación para Evitar la Ceguera en Mexico I.A.P., Luis Sánchez Bulnes Hospital in Mexico City, Mexico, in collaboration with the Vision Laboratory from the Instituto Politécnico Nacional, Mexico City, Mexico, from May 2015 to April 2017.

### 2.2. Patients

A total of 78 eyes from 46 patients with POAG were included in this study; 58 of these patients were receiving the maximum tolerated medical treatment and did not reach their target tensional values, whereas 20 patients were treatment naïve to drugs.

Key inclusion criteria included patients ≥40 years of age, diagnosed with POAG with or without topical treatment which required lowering the IOP to achieve their target IOP. We excluded patients with any previous ocular surgery, infectious eye disease, uveitis, or any other inflammatory ocular disorders, kwon allergies, corneal abnormalities, or previous conditions that prevent IOP measurement, as well as a history for ocular trauma.

All of the subjects had a comprehensive ocular examination including a review of the medical history, slit-lamp examination, Goldmann applanation tonometry, IOP measurement, central corneal thickness, cup to disk ratio, gonioscopy, optic nerve OCT, visual field evaluation, and dilated fundus examination.

### 2.3. Microstimulation Procedure

**Figure 1:**
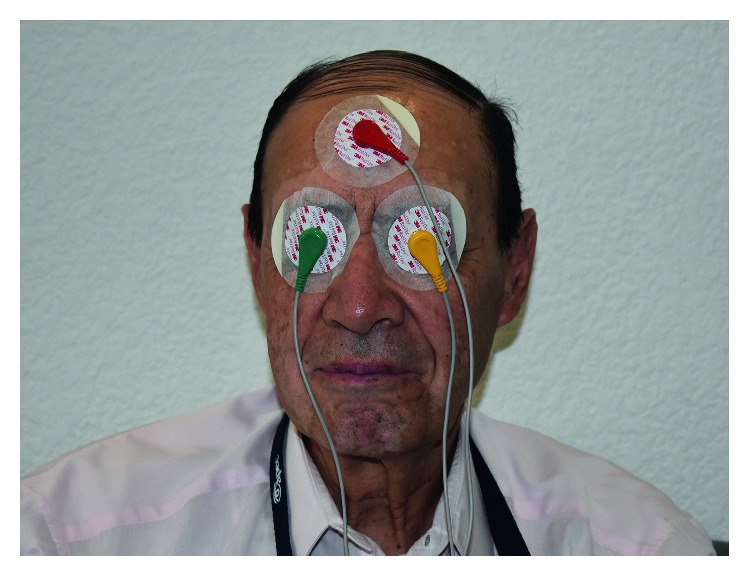
Transpalpebral electrical microstimulation procedure on a POAG patient.

The waveform more frequently used by authors in TES is shown in Figure 1. However, based on the antecedents previously discussed in Introduction, the tension response to electrical stimuli delivered by a transpalpebral device was studied in patients with glaucoma. This device reproduces electric profiles analogous to those reported by Stumpff and Wiederholt [10] in the BKCa^2+^ after the application of tyrosine kinase inhibitors and may be used as an adjuvant treatment to control IOP in patients with POAG. The waveform shown in Figure 1 fits more accurately with BKCa^2+^ ionic cells in TMC.

The electrical stimuli applied in our study consisted of 10 Hz, biphasic nonrectangular current pulses (up to 100 *μ*A) that were delivered from an isolated constant current stimulator. Every TPES application was performed at the same time of the day for all patients (between 9:00 and 10:00 h). The microstimulation procedure consisted of daily TPES application for 40 minutes attaching the current electrode to the eyelid employing polyurethane adhesive film fragments for a total of 10 days, divided into two 5-period days, with a rest of 2 days in between for a period of two weeks as depicted in Figure 1. Afterwards, application was continued twice per week for the remaining time until the four-month visit.

### 2.4. Instrumentation

**Figure 2:**
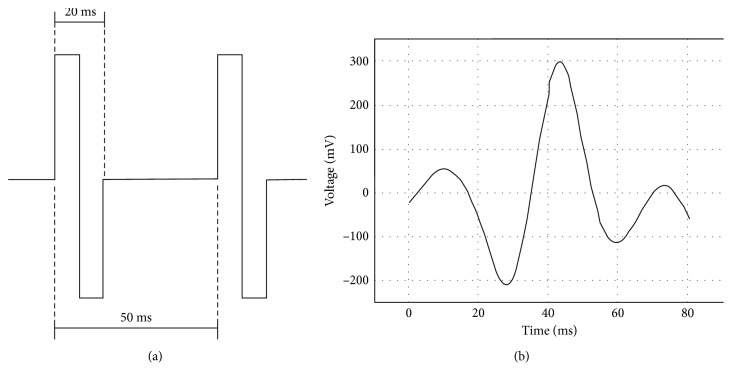
(a) Biphasic waveform applied on the most reported TES; (b) a new voltage waveform approach used in TPES.

The transpalpebral electronic stimulator based on a digital adaptive model delivers an electronic waveform generator specific for transpalpebral electrical stimulation. The applied waveform model is generated on a digital processor by means of an adaptive finite impulse response filter, in which the output waveform is synthesized form the original waveform registered from a multifocal electroretinograph as depicted in Figure 2.

### 2.5. Statistical Analysis

Statistical significance for IOP changes between groups was determined using a Student's *t*-test for normally distributed variables. Otherwise, we employed the Wilcoxon matched-pairs signed rank test. In addition, a Pearson correlation coefficient (*r*) and linear regression analysis were obtained between age and the fourth-month visit IOP (95% CI and a two-tailed *P* value). *P* values less than 0.05 were considered statistically significant. Normal and nonnormal distributions were determined using the Shapiro–Wilk tests for all variables.

Statistical analyses were performed using the Statistical Package for Social Sciences (SPSS) software (version 20, SPSS, Inc., Chicago, IL, USA). Graphs and layouts depicted in the Results were elaborated using the 2015 Graph Pad software Inc., Prism version 6.0.

## 3. Results

A total of seventy-eight eyes from 46 POAG patients were included in this study, 58 eyes with maximum tolerated medical treatment and 20 eyes without treatment (naïve). No statistically significant differences were evidenced between groups in terms of age (*P*=0.358). All demographic data are summarized in Table 1.

The mean baseline IOP on the maximum tolerated medical treatment group was 19.25 mmHg ± 4.71. On the contrary, the mean baseline IOP on the treatment-naïve patients was 20.38 mmHg ± 3.28. At the four-month follow-up visit, the mean IOP values obtained on the maximum tolerated medical treatment group was 14.41 mmHg ± 2.06, which accounts for a 25.14% IOP reduction. The obtained mean IOP measurement on the treatment-naïve group was 15.29 mmHg ± 2.28, which represents a 25.97% IOP reduction.

**Figure 3:**
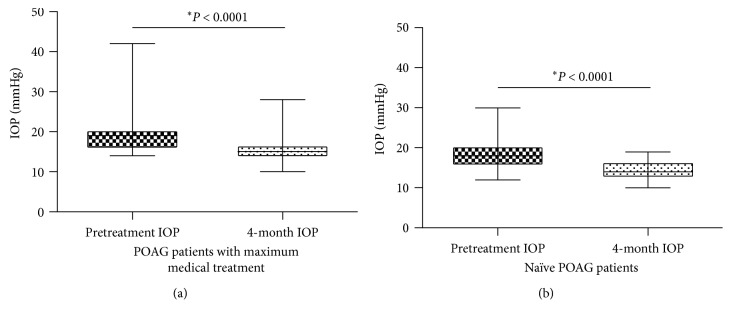
(a) Comparison between IOP values obtained before electrical stimulation to those measured at the four-month follow-up visit for POAG patients receiving the maximum tolerated medical treatment. (b) Comparison between IOP values obtained before electrical stimulation to those measured at the four-month follow-up visit from naïve patients. ^*∗*^Wilcoxon matched-pairs signed rank test.

In Figure 3, IOP comparison is depicted between baseline and four-month follow-up visit (*P* < 0.0001) for all POAG patients including those with maximum tolerated medical treatment (Figure 3(a)) and those without treatment (*P* < 0.0001) (Figure 3(b)).

**Figure 4:**
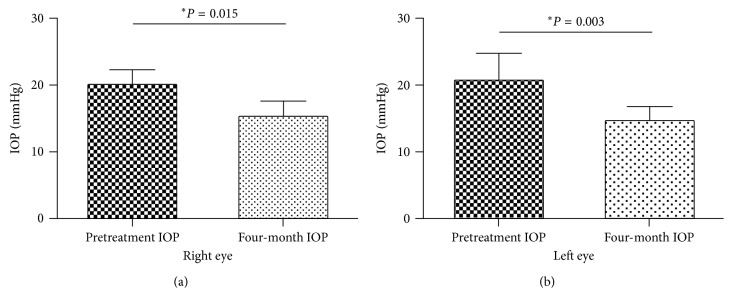
IOP measurements obtained from POAG patients without treatment (naïve): (a) comparison of IOP values before electrical stimulation to those obtained at the four-month follow-up visit for the right eye; (b) comparison of IOP values before electrical stimulation to those obtained at the four-month follow-up visit in the left eye. ^*∗*^Wilcoxon matched-pairs signed rank test.

In Figure 4, IOP comparison is described between baseline and four-month follow-up visit measurement for the right eye (Figure 4(a)) and the left eye (Figure 4(b)) on the naïve POAG patients.

**Figure 5:**
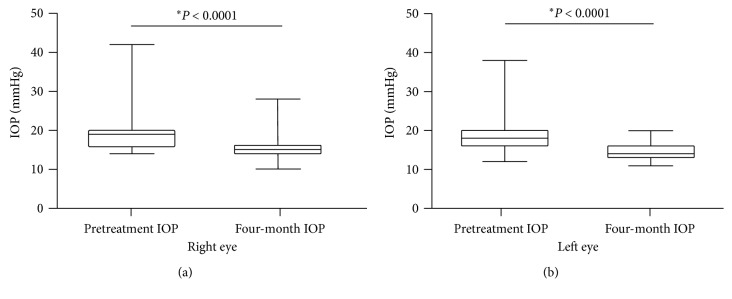
IOP measurements obtained from POAG patients receiving maximum tolerated medical treatment: (a) comparison between IOP values obtained before electrical stimulation to those obtained at the four-month follow-up visit for the right eye; (b) comparison between IOP values obtained before electrical stimulation to those obtained at the four-month follow-up visit for the left eye. ^*∗*^Wilcoxon matched-pairs signed rank test.

The compared IOP values for POAG patients with maximum tolerated medical treatment at baseline and at the four-month follow-up visit are presented in Figure 5.

**Figure 6:**
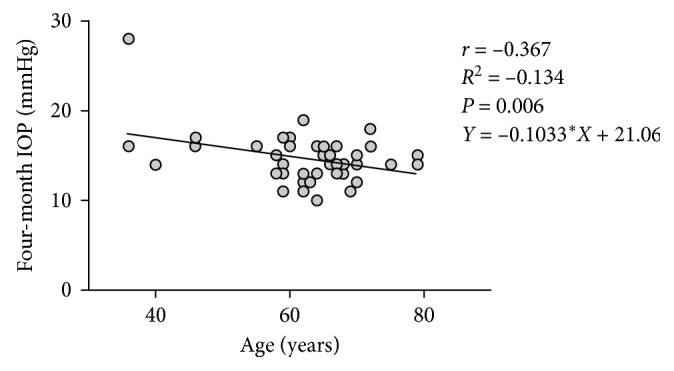
Coefficient of correlation (*r*) and linear regression analysis (*R*^2^) between POAG patients' years of age and IOP measurement at the four-month follow-up visit. ^*∗*^Pearson correlation coefficients (*r*), 95% CI, and two-tailed *P* value.

The Pearson correlation coefficient (*r*) and linear regression analysis (*R*^2^) obtained between patients' years of age and the final IOP measurement at the four-month follow-up visit were as follows: *r*=−0.367, *R*^2^=−0.134,  and *P*=0.006, as depicted in Figure 6.

## 4. Discussion

Understanding the physiology of the trabecular cell, the active membrane components that explain its activity, the pathophysiology of trabecular dysfunction, and the role of ion channels, primarily maxi K channels, facilitate an alternative therapeutic hypotensive ocular approach for glaucoma patients [10].

In recent years, the electrophysiology of TMCs has garnered increasing interest, and the behavior of voltage-dependent BKCa^2+^ channels and their relationship to various conditions, such as POAG, has been studied [11]. There are three types of voltage-dependent potassium channels in both healthy and glaucomatous cells. In the cases studied, the behavior of the ionic currents of 3 different K^+^ channels for normal TMCs and glaucomatous TMCs (GTMCs) exhibited significant differences in membrane amplitude and voltage [11].

We propose the application of transpalpebral specific exogenous voltages to act similarly to tyrosine kinase inhibitors. Specifically, applying an electric potential from the eyelid induces membrane potentials analogous to those found by Stumpff and Wiederholt [10] and Soto et al. [11] and stimulates the reactivation of BKCa^2+^ in TMCs. The application of exogenous electric fields can reactivate the BKCa^2+^ sequestered by the action of kinases in patients with POAG, obtaining an analogous reaction to that reported by tyrosine kinase inhibitors, thus contributing to IOP control [10–15].

Most reported research in TES, both indirect and direct stimulation, uses simple bipolar square waveforms to target a complex neural network system at the inner retina [8, 9]. Only a few authors suggest stimulation by sinusoidal and other nonsquare waveforms [14, 15]. Square bipolar waveforms are not consistent with the typical cell ionic current patterns. However, the relationship between TES parameters and the effect on IOP and other eye structures is still unclear.

In our study, the ocular hypotensive response obtained was statistically significant for both groups, the treatment-naïve group and the group of POAG patients with previous maximum tolerable treatment. Moreover, the results of the previously treated group clinically confirm the rescue of primary trabecular function in the setting of reduced AH production and the stimulation of the uveoscleral or nonconventional drainage pathways [14].

The hypotensive response obtained in this group of patients, even in patients having received maximum tolerable treatment, was comparable to that described with prostaglandin analogues or various drug combinations [16, 17]. Therefore, this treatment could be comparable to first-line ocular hypotensive treatment but does not affect the ocular surface or exert systemic or local side effects.

The flexibility to generate any desired action potential opens fresh opportunities to brand new experiments in transpalpebral stimulation. Our stimulator system can reproduce any real biological ionic currents with 99.9% accuracy. These new approaches to stimulate TMCs open brand new opportunities to understand more precisely the neuroregulation effects of electrical stimulation in glaucoma.

Transpalpebral electrical microstimulation is not an invasive procedure. Thus, this treatment modality is advantageous to conventional medical treatment consisting of hypotensive ocular drugs, which are associated with local and possible systemic side effects. This procedure induces no changes on the ocular surface in opposition to ocular surface changes induced by the topical medication and the preservatives. No inflammatory phenomenon is generated; moreover, the immunological apparatus remains unaltered. Electrical microstimulation focuses to the true cause of ocular hypertension, that is, the treatment of the dysfunctional trabeculum, while the rest of the therapy is focused on reducing the production of the aqueous humor or the exit of it through the unconventional route. In addition, it is important to emphasize the fact that this approach is highly cost efficient, especially when compared to the gold standard antiglaucoma therapy, which can elevate the long-term overall expenditures.

Long-term follow-up of larger cohorts will allow identifying potential refractory periods in the trabecular function or functional recovery of trabecular cells. Thus, such studies are necessary to demonstrate the effectiveness of this procedure.

## Figures and Tables

**Table 1:** Demographic data of included POAG patients.

Variable	Male	Female	Total	*P* value^*∗*^	95% CI of the difference
Eyes (*n*)	37	39	76	—	—
Gender (%)	48.69	51.31	100	—	—
Age (years)					
Mean ± SD	63.24 ± 11.12	59.05 ± 15.23	68.07 ± 12.06	0.358	−4.94 to 13.31
Range	40, 79	23, 77	12, 101		

^*∗*^Wilcoxon matched-pairs signed rank test.
